# Editorial: Multifaceted genes in amyotrophic lateral sclerosis-frontotemporal dementia volume II

**DOI:** 10.3389/fgene.2024.1420029

**Published:** 2024-05-10

**Authors:** Francesca Luisa Conforti, Alan Edward Renton, Henry Houlden

**Affiliations:** ^1^ Department of Pharmacy, Health and Nutritional Sciences, University of Calabria, Arcavacata di Rende, Italy; ^2^ Icahn School of Medicine at Mount Sinai, New York, NY, United States; ^3^ University College London, London, England, United Kingdom

**Keywords:** C9ORF72 ALS/FTD, FTD (frontotemporal dementia), ALS (amyotrophic lateral sclerosis), miRNA, microRNA, missence variant

Building on the first Volume of Multifaceted Genes in Amyotrophic Lateral Sclerosis-Frontotemporal Dementia, this new Research Topic includes papers focusing on the most common genetic cause of amyotrophic lateral sclerosis (ALS) and frontotemporal dementia (FTD) in European ancestry populations, the *C9orf72* hexanucleotide repeat expansion mutation. Broce et al. explored the neuroanatomical basis of shared genetic risk in the ALS-FTD spectrum by identifying genes exhibiting regional co-expression patterns similar to *C9orf72*. Aiming to elucidate why certain brain regions are susceptible to neurodegeneration in *C9orf72*-linked ALS-FTD, the authors uncovered a *C9orf72*-associated gene network that also tracked cortical thickness in *C9orf72* repeat expansion carriers. Finally, they showed that this network was enriched in brain cell populations and regions known to be selectively vulnerable in ALS-FTD, and investigated the molecular pathways involved.

Another interesting aspect concerns *C9orf72* intermediate-length repeat alleles in ALS-FTD. Their precise role remains unknown, but these alleles are much more frequent than the repeat expansion. Therefore, if they pose a disease risk, a significant proportion of ALS patients could benefit from treatments in development that target the *C9orf72* repeat expansion. In the Finnish population, Kaivola et al. identified a haplotype containing a subset of intermediate-length alleles that increases the risk of ALS. The authors also showed in the FinnGen biobank cohort that this haplotype reduces survival after the age of 80, which may have implications for neurodegenerative diseases other than ALS-FTD.

This Research Topic also focuses on other genetic loci contributing to the disorders. In particular, the study by Miura et al. reports a sporadic case of ALS-FTD carrying a p. Arg89Trp missense variant in the *VCP* gene. *VCP* genetic variants predicted to alter this amino acid residue have previously been found in patients diagnosed with FTD-distal myopathy and with ALS, further demonstrating that this gene can cause phenotypic pleiotropy. This underscores the importance of studying *VCP* genetic variants in patients with ALS and FTD, even in those without a family history of these diseases.

In addition, Brusati et al. highlighted the contribution of microRNA (miRNA) genetic variation to ALS. In this study, the authors developed a workflow to detect miRNA rare variants in ALS whole genome sequencing data and predict their biological effects on miRNA folding, maturation, and target gene recognition. This approach suggests that ALS-carriedmiRNA rare variantsimpact nervous system development, although these preliminary findings await confirmation and functional validation.

The four articles in this Research Topic expand our understanding of the genetic architecture and genotype-phenotype relationships of ALS-FTD. These findings help move the field forward as we progress toward precision medicine approaches to combat these devastating diseases.

